# BDNF Gene's Role in Schizophrenia: From Risk Allele to Methylation Implications

**DOI:** 10.3389/fpsyt.2020.564277

**Published:** 2020-12-15

**Authors:** Xiaoqian Fu, Jun Wang, Jianbin Du, Jing Sun, Ancha Baranova, Fuquan Zhang

**Affiliations:** ^1^Department of Clinical Psychology, Suzhou Guangji Hospital, The Affiliated Guangji Hospital of Soochow University, Suzhou, China; ^2^Department of Psychiatry, Wuxi Mental Health Center of Nanjing Medical University, Wuxi, China; ^3^Department of Psychiatry, The Affiliated Brain Hospital of Nanjing Medical University, Nanjing, China; ^4^School of Systems Biology, George Mason University, Fairfax, VA, United States; ^5^Research Centre for Medical Genetics, Moscow, Russia; ^6^Institute of Neuropsychiatry, The Affiliated Brain Hospital of Nanjing Medical University, Nanjing, China

**Keywords:** schizophrenia, BDNF, single nucleotide polymorphism, rs6265, methylation

## Abstract

**Background:** Schizophrenia (SZ) is a severe chronic mental disorder with complex genetic mechanisms. Brain-derived neurotrophic factor (BDNF) is one of promising candidate genes for SZ, and rs6265 is a non-synonymous single nucleotide polymorphism (SNP) in BDNF.

**Methods:** In this study, we performed a case-control association study of rs6265 in a cohort of Han Chinese population from eastern China, including 1,407 SZ patients and 1,136 healthy controls; and carried out a cis-mQTL (Methylation Quantitative Trait Loci) analysis for BDNF rs6265.

**Results:** We found a positive association of rs6265 with SZ (*P* = 0.037), with the minor allele (A) of rs6265 conferring a protecting effect for SZ (OR = 0.89). Furthermore, cis-mQTL analysis indicates that rs6265 is associated with several methylation loci surrounding BDNF.

**Conclusions:** Together, our findings provide further evidence to support the involvement of BDNF gene in the genesis of SZ.

## Introduction

Schizophrenia (SZ) is a serious mental disorder featured with profound disruption in emotion and cognition, affecting the most basic human properties such as language, thought, perception, and so on. The neurodevelopmental hypothesis is one of dominant hypotheses for SZ study in the last two decades, which supposes that SZ originates from developmental disorders of the nervous system early in brain development, long before the onset of the illness (1).

Brain-derived neurotrophic factor (BDNF) is believed to be involved in the pathophysiology of SZ and has been widely studied as a marker of neuropsychiatric diseases (2). The expression or functional changes of BDNF are proven associates of the pathophysiological process of several brain diseases, including mental diseases and neuro degenerative diseases (3). Animal studies have demonstrated the importance of BDNF in neurodevelopment and survival. Most of the homozygous BDNF mutant mice die within 2 days of birth, with some surviving 2 to 4 weeks. They exhibit distinct behavioral phenotypes as well as lack motor coordination and balance (4). BDNF affects cell level maturation, survival, diffusion, and synaptic function by activating intracellular signaling cascades, including mitogen-activated protein kinase/extracellular signal regulated protein kinase (MAPK/ERK), phosphatidylinositol 3-kinase, and phospholipase Cc pathways (5–7).

The single nucleotide polymorphism (SNP) rs6265 in *BDNF*, also known as Val66Met or G189A replacement at codon 66 in the pro-region of BDNF, alters the classification of BDNF protein and its availability in the synaptic cleft (2). The rs6265 variant interferes with the activity-dependent secretion of BDNF by inhibiting the sorting of BDNF into secretory granules, thereby affecting its function (8). An association of rs6265 polymorphism with the changes in hippocampal structure and function has been replicated in both human and mice (8, 9). The BDNF rs6265 knock-in mice show decreased BDNF expression, reduced hippocampal neurogenesis (10), decreased hippocampal volume, and abnormal morphology of hippocampal neurons (9). It has been suggested that rs6265 genotypes in the hippocampus and infra limbic medial prefrontal cortex may affect NMDA receptor-mediated neurotransmission and plasticity, which are associated with the production of positive or negative symptoms of schizophrenia (11, 12). Moreover, there is increasing evidence that rs6265 modifies both the clinical presentation and genetic risk architecture of schizophrenia, possibly by influencing cognitive function, brain morphology, age of onset, and treatment response (13, 14).

Prenatal stress is deemed to be a risk factor for SZ as a neurodevelopmental disorder (15). Dong et al. (15) reported both the SZ-like behavioral abnormalities in adult offsprings of mice exposed to prenatal stress mice and the molecular changes in the postmortem brains of SZ patients, with expression levels of DNA-methyltransferase 1 (DNMT1) and 10-11-translocation hydroxylase being significantly increased in the frontal cortex and hippocampus. Moreover, the corresponding reduction of *BDNF* transcription levels, along with enrichment of 5-methylcytosine and 5-hydroxymethylcytosine in the regulatory regions of *BDNF* gene, were observed, pointing at important role of *epi*genetic modification of *BDNF* in the phenotype and pathogenesis of SZ. It's worth noting that epigenetic modifications, including DNA methylation of the *BDNF* promoter, are significantly related to the pathophysiology of psychiatric disorders (4).

To further elucidate the role of the *BDNF* gene as a risk allele or regulator in SZ, we performed a case-control association study of rs6265 in a cohort of Han Chinese population from eastern China, including 1,407 SZ patients and 1,136 healthy controls, and carried out a cis-mQTL (Methylation Quantitative Trait Loci) analysis for BDNF rs6265.

## Materials and Methods

### Subjects

All subjects were unrelated Han Chinese recruited from China. In the patient group, the diagnosis of SZ was in line with criteria in the Diagnostic and Statistical Manual of Mental Disorders, Fourth edition (DSM-IV) and confirmed by two or more experienced psychiatrists using the Structured Clinical Interview for DSM-IV (SCID-I). Exclusion criteria included the presence of other mood or neurodevelopmental disorders, epilepsy, or intellectual disability. For the choice of healthy controls, the Structured Clinical Interview for DSM-IV, Non-patients edition (SCID-NP) was used to interview members of an unrelated general population and exclude those with mental illness by professional psychiatrists.

For genetic association analysis, our study sample includes 1,407 SZ patients (874 men and 533 women, aged 45.8 ± 11.5 years) and 1,136 healthy controls (633 men and 503 women, aged 44.9 ± 10.3 years). Healthy subjects were recruited through advertisement. This study was approved by the Ethics Committees of the Wuxi Health Mental Center, and either patients or their guardians signed informed consents.

### Genotyping

Peripheral blood samples were collected from all subjects. Blood samples were collected from all participants using K2EDTA tubes and a Blood Genotyping DNA Extraction Kit. The genotype of the SNP was analyzed by the Shanghai Biowing Applied Biotechnology Co. Ltd. (www.biowing.com.cn) using the Ligase Detection Reaction-Polymerase Chain Reaction method. Genomic DNA extracted from blood samples was first subjected to multiplex RCR to obtain a PCR product including SNPs. The PCR product and LDR probes were then subjected to multiplex LDR reaction, with a DNA sequencer to detect the products (1).

### Statistical and Bioinformatics Analysis

Genetic association tests were analyzed using PLINK v1.07 (16). The data obtained from SZ patients and healthy controls was compared. SNP association analyses were performed to test for possible associations between SNP rs6265 in BDNF and SZ using Plink v1.07. The two-tailed Fisher's exact test was used to compare the polymorphisms' distributions and testing their significance at *p* < 0.05 (17). The *p*-values were adjusted by false discovery rate correction for multiple test analysis (17). The allele frequencies and genotype distribution of rs6265 were calculated for the SZ cases and healthy controls and were analyzed for association by Logistic regression with the assumption of an additive genetic model, and Odds ratios (OR) with 95% confidence intervals were calculated (18). We performed the cis-mQTL in the methylation dataset (19) using Genevar 3.3 (20), with which we analyzed the association of rs6265 genotypes with neighboring methylations within 100 Kb distance. Associations between DNA methylation levels and probabilities of imputed genotypes were tested in samples of related individuals by a two-step analysis (19). That is, estimating a linear mixed model of methylation levels, covariates, and a kinship matrix, and then a score test. Age, beadchip, BS conversion efficiency, and BS-treated DNA input were cofactors. Cis analysis was limited to SNPs located within 100 kb of either side of the probe location and false discovery rate for the cis analysis was calculated with the q value package (19).

## Results

### Genetic Association

Genetic association analysis was performed in a study sample comprised of 1,407 patients (874 men and 533 women, aged 45.8 ± 11.5 years) and 1,136 unrelated healthy controls (633 men and 503 women, aged 44.9 ± 10.3 years).

In both the patient and the control groups, genotypic distributions of rs6265 had not deviated from Hardy-Weinberg equilibrium (HWE) (*P* > 0.05). Allelic distribution of rs6265 was associated with SZ (*P* = 0.037), with the minor allele (A) of rs6265 conferring a protecting effect for SZ (OR = 0.89). Specifically, OR = 0.89 indicates that minor allele (A) of rs6265 is negatively correlated with SZ, and reduces the risk of SZ in its carriers (Table 1).

**Table 1:** Genetic association of rs6265 with schizophrenia.

**Trait**	**A (freq)**	**G (freq)**	**OR (95%CI)**	***P***
Schizophrenia	1,339 (0.476)	1,475 (0.524)	0.89 (0.80–0.99)	0.037
Control	1,148 (0.505)	1,124 (0.495)		

*freq, frequency*.

### cis-mQTL Analysis

**Figure 1:**
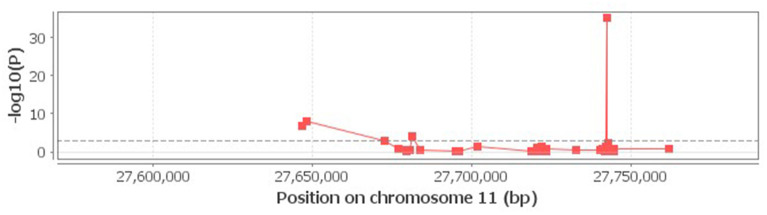
Correlation of rs6265 with neighboring methylations. The 4 loci above the dash lime are deemed to be significant (*P* < 0.001).

When the associations of rs6265 genotypes with neighboring methylations within 100 Kb distance were studied using the cis-mQTL analysis, an association of rs6265 with 4 methylation loci near or within BDNF was detected (Figure 1).

## Discussion

SZ is one of most disabling mental disorders; it affects the whole brain functions. Although the origin of this disorder remains unclear, a bulk of evidence supports that the abnormalities of early brain development play an important role in the pathogenesis of SZ.

In recent years, researches have focused on the effects of BDNF on brain development in the early stages of psychosis. For example, a significant correlation between the rs6265 polymorphism of *BDNF* and psychosis risk was found (21). An obvious association between the *BDNF* rs6265 genotype and the onset age of psychosis has also been demonstrated (22). Serum BDNF levels in first-episode psychosis patients were found to be much lower than those in the control group (23). Cumulatively, these findings indicate that BDNF may play an important role in the early stages of SZ.

For genetic association analysis, we found a positive association between rs6265 and SZ, with the minor A-allele of rs6265 conferring a protecting effect for SZ. A meta-analysis found that individuals with the Met/Met genotype had a 19% increased risk of developing SZ compared to individuals with the Val/Met genotype (24), a conclusion consistent with our results. However, it has been suggested that the non-mutated 66Val allele may be a risk locus for SZ, while the 66Met allele may actually be protective (14). For example, it was found that the Val gene conveyed risk in 321 Scottish SZ-spectrum probands (25). Moreover, a significant association was found between Val/Val genotype frequency and male patients with chronic SZ (26). The reasons for the inconsistencies in results may include differences in sample size, ethnic heterogeneity, etc., which still need further investigation.

A meta-analysis of imaging studies in neuropsychiatric patients with SZ, bipolar disorder, major depression or anxiety, showed that psychiatric patients with different BDNF rs6265 genotypes, such as Val/Val homozygous and Met-carrier both had smaller hippocampal volume as compared to that in healthy controls (2). Considering that rs6265 SNP was not associated with decreased hippocampal volume in neuropsychiatric patients, it followed that the Met allele might not be a risk allele (A/Met) for SZ (2). This hypothesis supports our findings pointing that allele A of rs6265 confers a protective effect for SZ. Due to a wide range of psychiatric disorders included in the meta-analysis cited above, whether this conclusion holds for the association between SZ and rs6265 genotype required further investigation.

At present, the body of literature describing results of the risk association studies of rs6265 and SZ remain contradictory due to lack of strong evidence and small sample sizes (14, 27). In view of larger sample size (1,406 SZ patients and 1,136 healthy controls), our findings may be important for bringing more clarity to the association between rs6265 and SZ.

cis-mQTL analysis indicated an association of rs6265 with several methylation loci within *BDNF*. As our study strongly suggests that rs6265 is associated with SZ, mQTL findings may be used to support the role of BDNF related methylation in the etiology of SZ. The peripheral blood level of DNA methylation of *BDNF* in SZ were accessed previously, with more methylated alleles and lower expression levels of BDNF found in the patient group than in the control group (28). Our findings are consistent with those observation, suggesting that BDNF related methylation may play an important role in SZ. Ikegame et al. (4) put forth that the down-regulation of *BDNF* levels is commonly associated with the increase in DNA methylation of the *BDNF* promoter. Cell and animal models have shown that the expression of BDNF in the neurons may be regulated by DNA methylation of specific promoters, but the mechanism of elevated DNA methylation at those specific sites is still elusive (4).

One previous study investigated peripheral blood lymphocytes of SZ patients and found the changes in DNA methylation of *BDNF* promoter I, thus, connecting the epigenetic alteration of *BDNF* locus in peripheral blood cells to the pathophysiology of SZ (29). Dong et al. (30) have shown that mice born from dams stressed during pregnancy develop behavioral deficits similar to those detected in adult SZ patients. Moreover, they showed that clozapine treatment reverses both the behavioral deficits and 5-methylcytosine and 5-hydroxy methylcytosine changes at *BDNF* promoters, as well as reduces mRNA and protein expression of this gene (30). This provides further evidence that BDNF-related methylation may play an important role in the pathophysiology of SZ.

On the other hand, in a study of the prefrontal cortex samples from 25 SZ patients and 25 healthy controls, the risk of SZ was independent of *BDNF* mRNA expression levels and the differences in DNA methylation of its promoter (31). Another study examined the DNA methylation levels of *BDNF*-encoded exons and three different promoter regions in the prefrontal cortex of SZ patients and found no significant differences in DNA methylation in patients with schizophrenia when their brain regions were compared to that of the controls (32). In sum, the above studies did not find an association between *BDNF* and DNA methylation in SZ. Since these studies were made using samples from the prefrontal cortex of the postmortem brains, these conclusions are vulnerable to possible bias due to their postmortem character, precluding direct comparisons with the conclusions made using living samples from the cells peripheral blood.

Kundakovic et al. (33) have explored a model of environmental exposure to bisphenol A in pregnant mice. Persistent changes of DNA methylation were detected in regions related to the transcription of *BDNF* gene in the hippocampus and blood of exposed pups. These changes were consistent with those in human umbilical cord blood of the newborns of exposed mothers, suggesting that the DNA methylation of *BDNF* in blood may serve as indicator for the DNA methylation of *BDNF* in the brain and the biomarker of behavioral vulnerability. Here we studied first-episode SZ patients and found an association between *BDNF* polymorphism rs6265 and methylation within *BDNF* locus. Our findings suggest that abnormal methylation of *BDNF*-related regions occurs early in SZ, pointing at the utility of these minimally-invasive for the detection of early SZ.

Many studies have found that epigenetic abnormalities in the SZ postmortem brain and the peripheral blood lymphocytes of SZ patients parallel each other. For example, in both types of samples, activities of cytosine modify DNMT1 and ten-eleven methylcytosine dioxygenase 1 increase, while amounts of BDNF-encoding mRNA, which is highly sensitive to the levels of its DNA methylation, decrease (34–41). In addition, studies have shown that, in SZ patients, increased DNMT expression may be seen as a probable cause of the concomitant decrease in *BDNF* expression, mediated by the DNMT-dependent elevation of cytosine methylation levels in *BDNF* promoter (36, 42). Davies et al. (43) showed that the DNA methylation patterns in the brain and the blood are highly correlated, including that in genes related to neural differentiation and neurodevelopment, such as BDNF. Furthermore, a similarity between brain- and lymphocyte-specifics changes of DNMT and ten-eleven methylcytosine dioxygenase 1 activities was shown (34, 44). In the case of SZ, the mechanisms of epigenetic regulation in the peripheral blood lymphocytes and the brain may be similar (34, 41, 43, 45). Therefore, the methylation levels in certain genes expressed in cells of the peripheral blood may be used as proxy biomarkers for early identification of SZ, and early interventions.

There are several limitations to consider in our study. The methylation data were derived from peripheral tissues rather than the brain itself, thus caution is needed when extrapolating the conclusions. Said that, detected abnormalities in peripheral tissue may serve as potential indicators of disease pathology even though they distinct from that in the brain. The changes in peripheral biomarkers may parallel pathological processes in the brain only in part, while in other part they would reflect the molecular reaction of the peripheral cells, which is secondary to the disease (46). Because both of these regulatory arms are reflective of the disease, “cause and consequences” arguments have lower applicability to biomarkers, which typically serve as indicators of association rather that causality. This study highlighted methylation biomarkers in the BDNF promoter as possible contributors for SZ detection in first-episode patients.

## Conclusion

Our study supports the association between BDNF polymorphism rs6265 and SZ, as well as the relevance between rs6265 and BDNF methylation, providing further evidence to support the involvement of BDNF gene in the genesis of SZ.

## Data Availability Statement

The datasets presented in this study can be found in online repositories. The names of the repository/repositories and accession number(s) can be found below: European Variation Archive, accession no: PRJEB41532 and ERZ1685357.

## Ethics Statement

The studies involving human participants were reviewed and approved by Ethics Committees of the Wuxi Health Mental Center. The patients/participants provided their written informed consent to participate in this study.

## Author Contributions

FZ designed the study and performed data analyses. XF, JW, JD, JS, and AB were responsible for manuscript writing and modification. All authors reviewed and approved the final manuscript.

## Conflict of Interest

The authors declare that the research was conducted in the absence of any commercial or financial relationships that could be construed as a potential conflict of interest.
